# Photocatalytic activity of the biogenic mediated green synthesized CuO nanoparticles confined into MgAl LDH matrix

**DOI:** 10.1038/s41598-024-52547-w

**Published:** 2024-01-28

**Authors:** Hildana Tesfaye Berede, Dinsefa Mensur Andoshe, Noto Susanto Gultom, Dong-Hau Kuo, Xiaoyun Chen, Hairus Abdullah, Tadele Hunde Wondimu, Yi-nan Wu, Osman Ahmed Zelekew

**Affiliations:** 1 Department of Materials Science and Engineering, Adama Science and Technology University, Adama, Ethiopia; 2 Department of Materials Science and Engineering, National Taiwan University of Science and Technology, Taipei, 10607 Taiwan; 3 College of Materials Engineering, Fujian Agriculture and Forestry University, Fuzhou, 350002 China; 4 College of Environmental Science and Engineering, State Key Laboratory of Pollution Control and Resource Reuse, Tongji University, 1239 Siping Rd., Shanghai, 200092 China; 5 Shanghai Institute of Pollution Control and Ecological Security, 1239 Siping Rd., Shanghai, 200092 China

**Keywords:** Environmental sciences, Chemistry, Materials science

## Abstract

The global concern over water pollution caused by organic pollutants such as methylene blue (MB) and other dyes has reached a critical level. Herein, the *Allium cepa* L. peel extract was utilized to fabricate copper oxide (CuO) nanoparticles. The CuO was combined with MgAl-layered double hydroxides (MgAl-LDHs) via a co-precipitation method with varying weight ratios of the CuO/LDHs. The composite catalysts were characterized and tested for the degradation of MB dye. The CuO/MgAl-LDH (1:2) showed the highest photocatalytic performance and achieved 99.20% MB degradation. However, only 90.03, 85.30, 71.87, and 35.53% MB dye was degraded with CuO/MgAl-LDHs (1:1), CuO/MgAl-LDHs (2:1), CuO, and MgAl-LDHs catalysts, respectively. Furthermore, a pseudo-first-order rate constant of the CuO/MgAl-LDHs (1:2) was 0.03141 min^−1^ while the rate constants for CuO and MgAl-LDHs were 0.0156 and 0.0052 min^−1^, respectively. The results demonstrated that the composite catalysts exhibited an improved catalytic performance than the pristine CuO and MgAl-LDHs. The higher photocatalytic performances of composite catalysts may be due to the uniform distribution of CuO nanoparticles into the LDH matrix, the higher surface area, and the lower electron and hole recombination rates. Therefore, the CuO/MgAl-LDHs composite catalyst can be one of the candidates used in environmental remediation.

## Introduction

Currently, environmental pollution as a result of the discharging of harmful and toxic organic pollutants from different sectors such as textile, plastic, paper, leather, and other industries become a current issue and a great challenge to mankind^1^. The discharging of these toxic organic pollutants into water bodies could be a threat to aquatic life and affect human health due to their carcinogenic and mutagenic properties^2,3^. Due to these reasons, different strategies have been implemented for the removal of organic pollutants^4,5^. However, the removal of organic pollutants with conventional treatment methods is not effective due to the chemical stability, complex structure, and low biodegradability of organic pollutants^6,7^. Specially, MB is one of the most commonly used dyes which makes mankind difficult to breathe, experiences vomits, and headaches^8^. Hence, it is crucial to design effective strategies and employ advanced treatment procedures that are useful for the complete removal of organic contaminants from polluted water systems^9^. One of the strategies employed for wastewater treatment is the advanced oxidation process (AOP) with a photocatalytic degradation system^10^. For this purpose, various photocatalysts such as TiO_2_^11^, ZnO^12^, Fe_2_O_3_^13^, CaO^14^, and CuO^15^ have been investigated. Among them, CuO has been widely utilized as a photocatalyst due to its excellent photovoltaic properties, robust electro-conductivity, and low band gap^16,17^.

Several physical and chemical methods have been utilized to synthesize CuO-based photocatalysts. However, most of the methods have limitations and require hazardous chemicals as a reducing agent^18,19^. To solve the limitations, a greener, cheaper, environmentally-friendly, simple, and non-toxic biological route has been applied to fabricate CuO NPs. In addition to this, using plant extracts in the synthesis of nanoparticles is considered as a potential candidate due to simplicity, large-scale production, and easy handling owing to natural reducing and capping agents^20^. For this purpose, several studies have been reported in the fabrication of CuO using biogenic-mediated strategies such as *Solanum lycopersicum*^21^, *Solanum nigrum*^8^, and *Psidium guajava*^22^*. *However, the biosynthesis of CuO NPs using the *Allium cepa* L. peel extract combined with LDHs has not been reported. Based on the above considerations, utilization of the *Allium cepa L* peel extracts in the preparation of nanoparticles is useful since it contains high concentrations of flavonoids and polyphenol compounds which are used as natural reducing and capping agents^23^.

Although CuO is a preferable semiconductor in photocatalytic applications, its activity is limited due to the higher electron and hole pairs recombination rates^24^. To improve the photogenerated electron and hole pairs separation efficiencies, different strategies such as doping^25^, heterojunction formation^26^, or combined with supports^27^, etc. have been implemented. Among the strategies, using support in the fabrication of catalysts showed higher catalytic performance than unsupported catalysts due to the interaction of organic pollutants on the surface of support via adsorption and reduction in electron–hole recombination rates^28^. Due to these reasons, the research works with different supports such as graphene oxide^29^, carbon nanotubes^30^, and layered double hydroxides (LDHs)^31^ have been reported.

LDHs are hydrotalcite-like and inorganic layered substances made up of positively charged brucite-like host layers and charge-balancing interlayer anions. Their large surface areas make them ideal catalysts or precursors^32^. The LDHs are commonly employed in catalysis, adsorption, electrochemistry, and biotechnology as typical two-dimensional-nano structured anionic clays^33^. Apart from the multiple positive charges on the LDHs platelets, the heterojunction interface has enough interlayer water molecules and hydroxide ions to form ·OH. The layer structure, in particular, may improve the effectiveness of photocatalytic processes by encouraging electron transport, inhibiting electron and hole recombination rates, and preventing nanoparticle aggregations^34^. Due to their superior properties, LDHs were deemed an appropriate substrate for growing nanoparticles to achieve synergistic effects and improved properties. Among them, the binary layered double hydroxides with magnesium and aluminum in the laminate have been widely used in photocatalysis^35^. However, the MgAl-LDH alone has limited photocatalytic activity under visible light irradiation. For these reasons, different composites such as TiO_2_/MgAl-LDH^36^, SnO_2_/MgAl-LDH^37^, and CeO_2_/MgAl-LDH^38^ have been reported.

Herein, a green approach using *Allium cepa* L. peel extract has been applied for the synthesis of CuO NPs and confined into the MgAl-LDH matrix for photocatalytic applications. The LDH layered structure could be used for effective electron migrations and electron–hole pair recombination rate suppression. The large number of hydroxyl groups on LDH laminates can also generate ·OH species, which is useful for boosting the photocatalytic activity and quantum efficiency^39^. Moreover, the green synthesized CuO nanoparticles could be also effective due to the wider visible light absorption ranges. It is expected that the composite catalysts could have improved performance than the single components due to the uniform dispersion of CuO NPs, high adsorption capacity of LDH, higher surface area, and lower electron and hole recombination rates.

## Experimental

### Chemicals

All chemicals and reagents were analytic grades and used without further purifications. Copper nitrate trihydrate (Cu(NO_3_)·3H_2_O, 95%), magnesium chloride hexahydrate (MgCl_2_·6H_2_O, 99%), aluminum nitrate nonahydrate (Al(NO_3_)_3_·9H_2_O, 98%), sodium bicarbonate (NaHCO_3_, 99.5%), sodium hydroxide (NaOH, 98%), ethanol (CH_3_CH_2_OH, 99.5%), hydrochloric acid (HCl, 37%), ethylenediamine tetra-acetic acid disodium salt (EDTA-2Na), isopropanol (IPA), and silver nitrate (AgNO_3_) were used. The fresh onion peels (*Allium cepa* L.) were collected from a cafeteria of Adama Science and Technology University. DI water was used throughout the experiment.

### Preparation of plant extract

The *Allium cepa* L. peel plant extraction process was performed according to literature report with modification^40^. In a particular procedure, the *Allium cepa* L. peels were washed with distilled water several times before being dried at 85 °C. The dried *Allium cepa* L. peels were then cut into small pieces. Then, five grams of the peel was transferred to a flask and 350 mL of distilled water was added. The mixture was stirred and boiled at 70 °C for 30 min to get an aqueous extract. Then, the solution was cooled at room temperature and filtered through filter paper.

### Synthesis of CuO nanoparticles (CuO NPs)

In the preparation of CuO NPs, copper nitrate trihydrate (Cu(NO_3_)·3H_2_O), sodium hydroxide (NaOH), and *Allium cepa* L. peel extract were used based on the literature report with minor modifications^22^. Specifically, 1.208 g of Cu (NO_3_)_2_·3H_2_O was dissolved into 100 mL of distilled water. Then, the resulting solution was dropped into an aqueous extract of *Allium cepa* L. peel (100 mL) under stirring for 45 min at room temperature. Then, aqueous solution of NaOH (0.5 mol L^−1^) was added to attain a pH of 10. The entire reaction was carried out at 70 °C for 3 h with constant stirring. The solution was aged for 24 h before being centrifuged and dried overnight at 80 °C. Finally, the powder sample was calcined at 400 °C for 2 h.

### Preparation of MgAl-LDHs

The MgAl-LDHs preparations were carried out using a co-precipitation method with Mg^2+^/Al^3+^ molar ratio of 3:1 according to a literature report with modification^41^. Briefly, an aqueous solution containing NaOH (4 g) and NaHCO_3_ (6.30 g) was added drop-wise to 100 mL of salt solution containing MgCl_2_·6H_2_O (0.015 mol) and Al(NO_3_)_3_·9H_2_O (0.005 mol) and stirred at 60 °C in water bath. The pH was set to 10.5 and the reaction was performed under continuous stirring for 2 h. The resulting precipitate was extensively rinsed with deionized water until the pH was neutral, after being aged at 60 °C for 12 h. The solid residue was collected and dried at 80 °C for 12 h.

### Preparation of CuO/MgAl LDHs nanocomposites

**Scheme 1:**
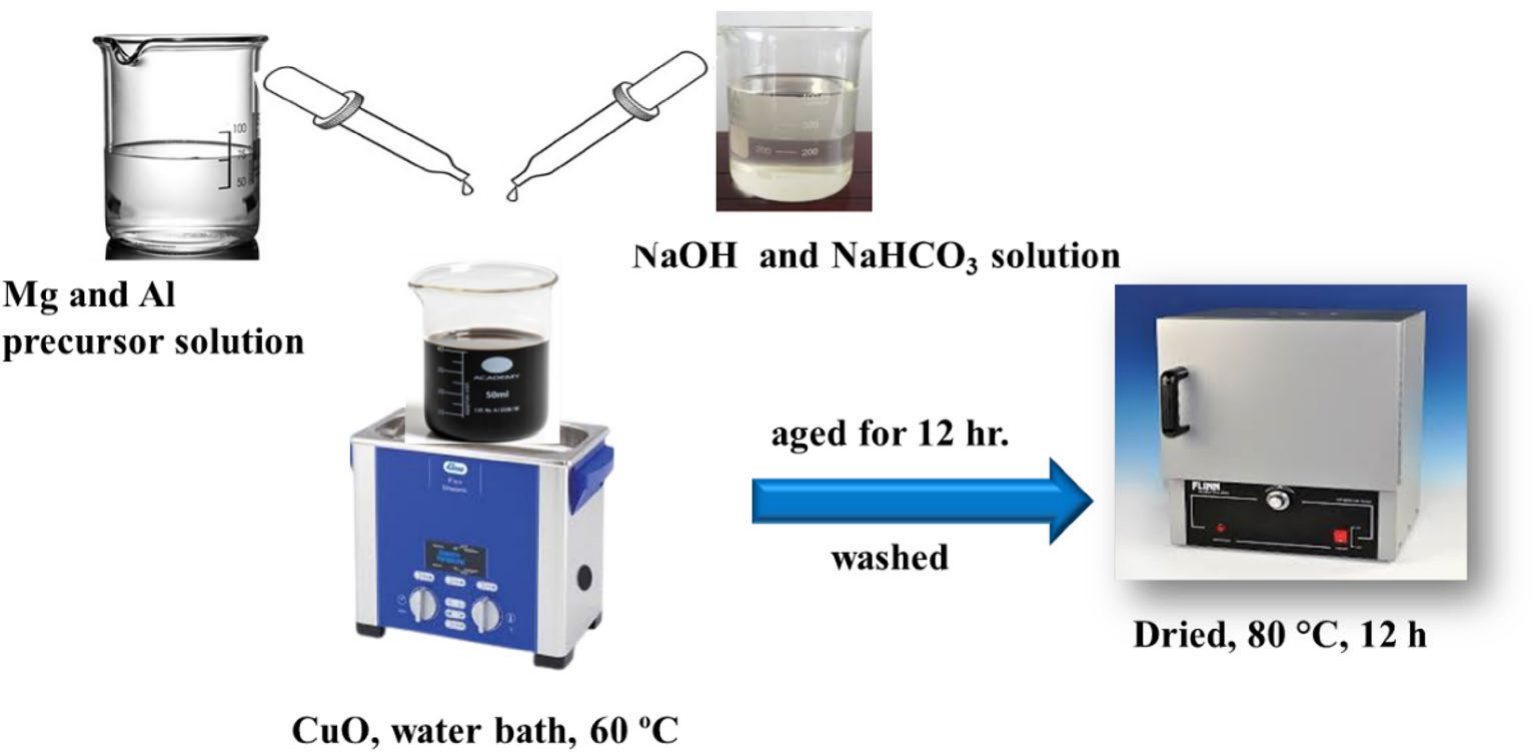
Schematic diagram for the synthesis of CuO/MgAl-LDHs composite catalyst.

Co-precipitation was used to prepare the CuO/MgAl-LDHs (1:2) composite catalyst. To obtain a uniform suspension, the prepared CuO (0.3077 g) was ultrasonically dispersed in 100 mL distilled water for 1 h and then placed in a 60 °C water bath with stirring. Then, a salt solution containing 0.192 g MgCl_2_·6H_2_O and 0.2424 g Al(NO_3_)_3_·9H_2_O were added to the suspension. To keep the pH at 10.5, an aqueous solution containing NaOH (4 g) and NaHCO_3_ (6.30 g) was added drop-wise. The precipitate was aged at 60 °C for 12 h and then washed with deionized water until the solution became neutral. Finally, the composite was dried for 12 h at 80 °C^42^. For comparison purposes, different weight ratios of the CuO/LDHs abbreviated as CuO/MgAl-LDHs (1:2), CuO/MgAl-LDHs (1:1), and CuO/MgAl-LDHs (2:1) were prepared. The preparation of the composite catalyst is shown in Scheme 1.

### Photocatalytic activity

The photocatalytic activities of the catalysts were checked by the degradation of MB dye. In the degradation process, 25 mg of the catalyst was added into 100 mL (10 mg L^−1^) of MB dye solution and stirred for 30 min to establish an adsorption–desorption equilibrium in the dark. Then, the suspension was exposed to a 150 W halogen lamp for 80 min under regular stirring. To analyze the degradation of the dye, 5 mL of the aliquot was taken every 20 min of reaction time followed by centrifugation. The absorbance at a maximum wavelength of 664 nm was used to determine the concentration of the dye leftover. The radicals scavengers effects in the degradation process of MB dye were performed and EDTA-2Na, isopropyl alcohol, and AgNO_3_ were used for trapping of h^+^, ·OH, and e^−^, respectively^43,44^. The stability of the CuO/MgAl-LDHs (1:2) catalyst was also checked according to the literature report with modification^45^. After each cycle, the catalyst was recovered by centrifugation, washed with deionized water, and dried in an oven at 80 °C.

### Characterizations

X-ray diffractometer (Shimadzu XRD-7000) with Cu Kα radiation (λ = 1.5406 Å) operating in the range of 2θ = 10° to 80, 30.0 mA applied current, and 40.0 kV acceleration voltage was used to check the crystallinity and phases of the samples. The average crystallite size (D) and interplanar spacing (d) were calculated according to Debye–Scherrer equation and Bragg’s Law, respectively^46,47^. Fourier transforms infrared spectroscopy (FTIR-6600 type A) was used to check bonding and functional groups of the samples in the range 4000–400 cm^−1^. The morphologies of the samples were examined by field-emission scanning electron microscopy (FE-SEM, JSM 6500F, JEOL) and transmission electron microscopy (TEM) (FEG TEM Tecnai G2 F30). X-ray photoelectron spectroscopy (XPS) (ESCALAB 250) was used to examine the chemical states of the sample. The JASCD FB-8500 photoluminescence (PL) spectroscopy was used to check the electron and hole separation rates. A Shimadzu-3600 plus UV–vis spectrophotometer was used to analyze the concentration of MB dye at a maximum wavelength of 664 nm. JASCD V-670 UV–visible–near-infrared (UV–vis–NIR) spectrophotometer was used to measure UV–vis diffuse reflectance spectra (DRS) using BaSO_4_ as a reference.

## Results and discussion

### Characterizations

**Figure 1:**
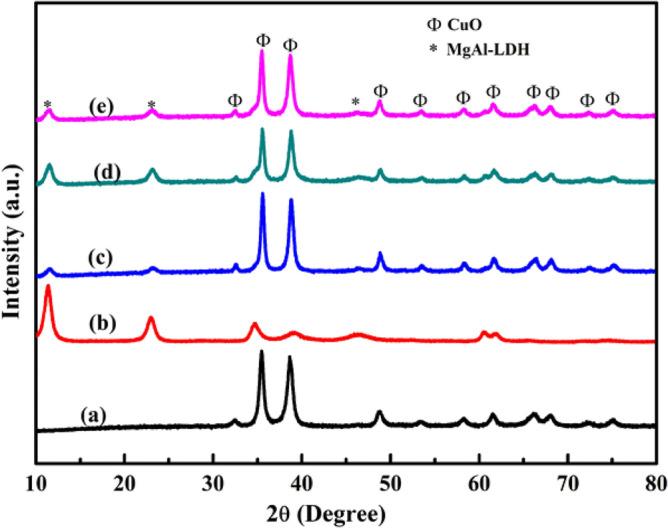
XRD patterns for: (**a**) CuO NPs, (**b**) MgAl-LDHs, (**c**) CuO/MgAl-LDHs (2:1), (**d**) CuO/MgAl-LDHs (1:2), and (**e**) CuO/MgAl-LDHs (1:1).

**Figure 2:**
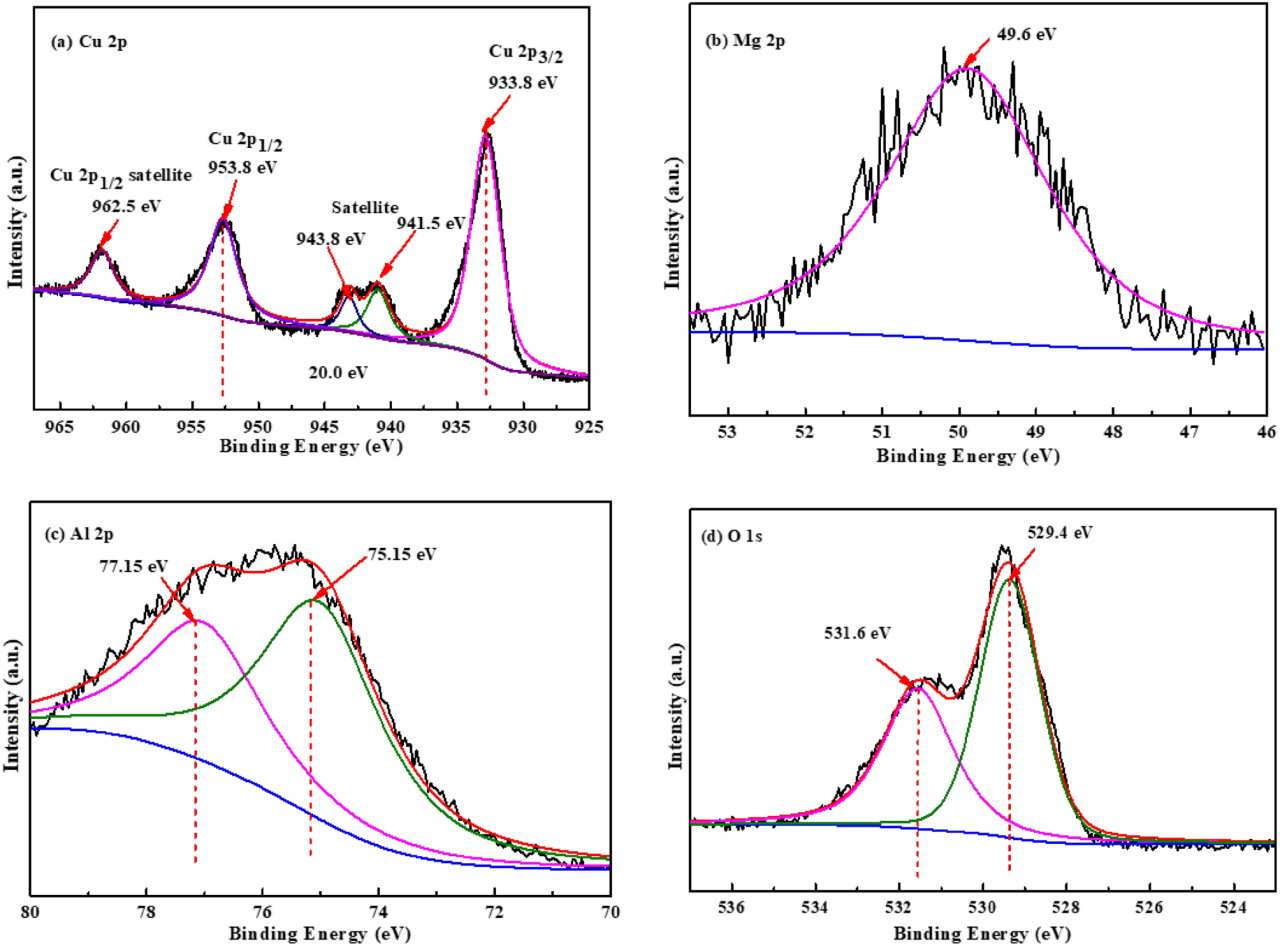
XPS spectra of (**a**) Cu 2p, (**b**) Mg 2p, (**c**) Al 2p, and (**d**) O 1s for CuO/MgAl-LDHs (1:2) sample.

The XRD patterns of the CuO NPs, MgAl-LDHs, and CuO/MgAl-LDHs composites with different weight ratios such as CuO/MgAl-LDHs (1:2), CuO/MgAl-LDHs (1:1), and CuO/MgAl-LDHs (2:1) are shown in Fig. 1. The characteristic peaks at 32.42°, 35.47°, 38.68°, 48.76°, 53.43°, 58.16°, 61.51°, 66.07°, 67.89°, 72.29°, and 75.00° correspond to the crystal planes of (110), ($\overline{1 }$11), (111), (20$\overline{2 }$, (020), (202), (11$\overline{3 }$, (31$\overline{1 }$), (113), (311), and (004), respectively, for CuO (Fig. 1a). The existence of all peaks correspond to the monoclinic CuO phase which is consistent with the reported research (JCPDS No. 00-048-1548)^48^. Moreover, the average crystallite size for CuO was also calculated and showed 16.1 nm with an interplanar spacing of 0.23 nm. Furthermore, as shown in Fig. 1b, the MgAl-LDHs peaks at 2θ of 11.4° and 23.0°, respectively, are matched with (003) and (006) crystal planes of Mg_6_Al_2_(OH)_18_·4.5H_2_O (JCPDS No. 22-0700). The phases are attributed to the hydrotalcite-type materials in a hexagonal lattice with R-3m rhombohedral symmetry which confirms the successful preparation of LDHs^49^. The major peaks at 2θ values of 11.44°, 23.03°, 34.75°, 39.06°, 46.41°, 60.54°, and 61.78° correspond to the crystal planes of (003), (006), (009), (012), (018), (110), and (113), respectively^50^. The average crystallite size for MgAl-LDHs was 7.95 nm with the interparticle spacing 0.39 nm. Moreover, the CuO/MgAl-LDHs XRD peaks clearly displayed the distinct peaks corresponding to both the CuO NPs and MgAl-LDHs. The results indicated that successful incorporation of CuO into LDHs was achieved. However, the prominent peaks observed confirmed the predominance of CuO in the sample. The presence of MgAl-LDHs as a carrier did not affect the structure of CuO NPs, as shown in Fig. 2c–e^39^. The average crystallite sizes were also calculated and showed 15.35, 13.9, and 12.5 nm for CuO/MgAl-LDHs (1:1), CuO/MgAl-LDHs (2:1), and CuO/MgAl-LDHs (1:2) samples, respectively. The results demonstrated that successful incorporation of CuO into LDHs was achieved.

The XPS analysis was used to investigate the chemical states of the elements in the CuO/MgAl-LDHs (1:2) composite sample. The peaks located at 933.8 and 953.8 eV, correspond to Cu 2p_3/2_ and Cu 2p_1/2_, respectively, which indicates the presence of Cu^2+^ in the CuO (Fig. 2a). Furthermore, the broad satellite peaks at a higher binding energy provided additional confirmation of the CuO. The Cu 2p was accompanied by two satellite peaks on the higher binding energy side at 943.8 eV and 941.5 eV, indicating the presence of CuO^51^. Moreover, the satellite peak at around 943.8 eV clearly demonstrates an open 3d^9^ shell, thus, supporting the presence of Cu^2+^ in the sample, which is positioned at higher binding energies than the main peaks^52,53^. Moreover, the high-resolution spectrum of the Mg 2p is also presented in Fig. 2b. In the Mg 2p spectra, a single peak appears at 49.6 eV, corresponding to Mg^2+^ in the brucite layers of MgAl-LDHs^54^. The Al 2p spectrum, deconvoluted into two peaks at 75.15 and 77.15 eV, can be assigned to Al 2p (α) and Al 2p (β) of Al(OH)_3_ and Al_2_O_3_, respectively, emerging from the LDHs structure, as shown in Fig. 2c^55^. Similarly, the binding energy at 529.4 eV belongs to lattice oxygen in the O 1s spectrum, while the peak at 531.6 eV reflects chemisorbed oxygen and coordinated lattice oxygen (Fig. 2d)^56^.

**Figure 3:**
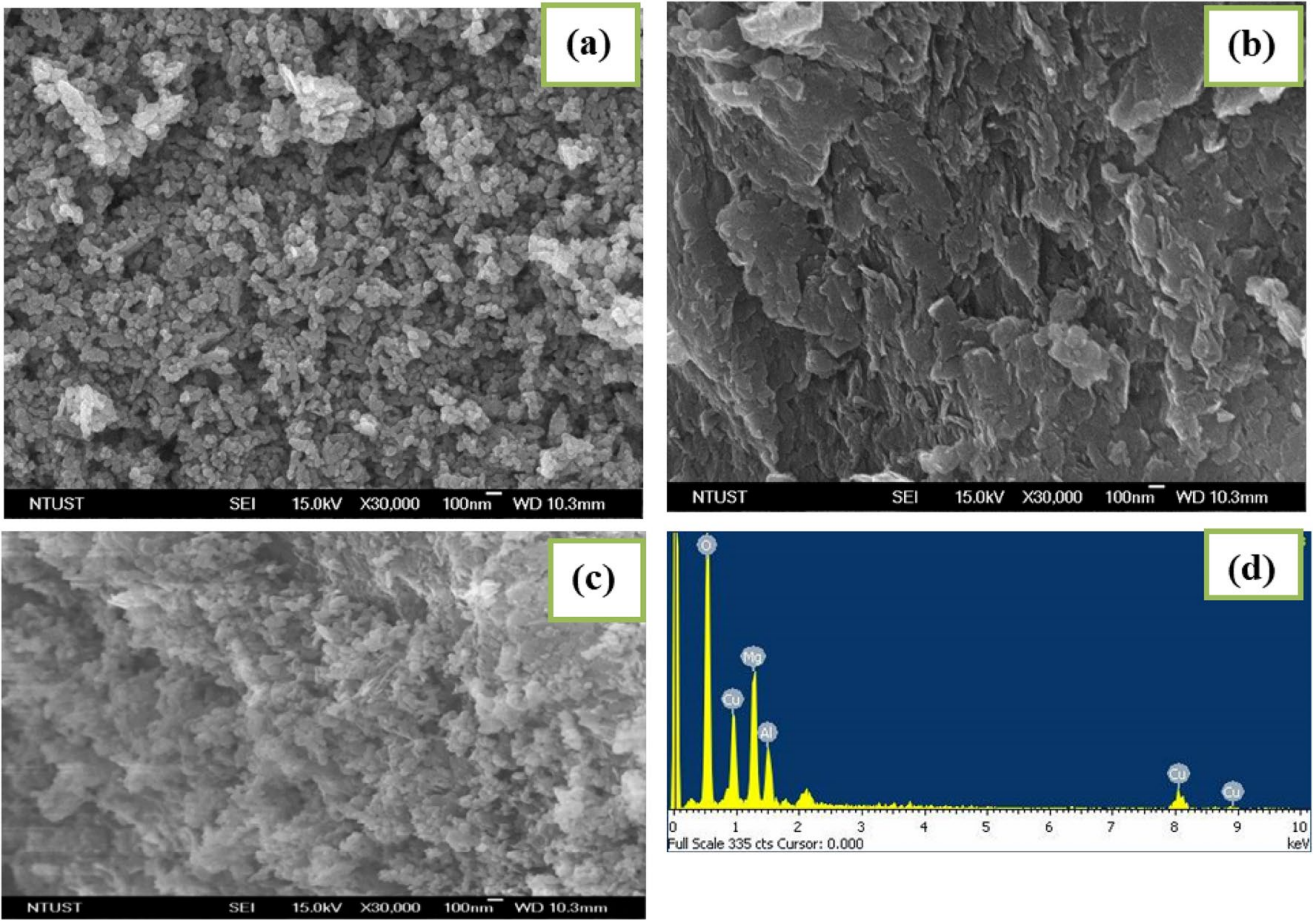
SEM images of: (**a**) CuO NPs, (**b**) MgAl-LDHs, and (**c**) CuO/MgAl-LDHs (1:2), and (**d**) EDS spectra for CuO/MgAl-LDHs (1:2) samples.

The FE-SEM was used to evaluate the morphological properties of the CuO NPs, MgAl-LDHs, and CuO/MgAl-LDHs (1:2) samples as shown in Fig. 3a–c. As it was observed in Fig. 3a, the CuO NPs had a spherical-like morphology. The biogenic-mediated preparation makes the CuO nanoparticles smaller and spherical morphology with different sizes. The smaller particles agglomerate and organize themselves into larger spheres^57^. In the preparation, the presence of functional groups in the plant extract encourages dynamic behavior during nucleation and stabilization^58^. Figure 3b displays the sheet-like structure of the prepared MgAl-LDHs. Moreover, Fig. 3c, showed the SEM image of the CuO/MgAl-LDHs (1:2) sample. The image reveals the uniform and dense distribution of CuO NPs in the MgAl-LDHs. The elemental composition of the as-synthesized catalyst was also analyzed through the EDS analysis (Fig. 3d). The EDS spectrum of CuO/MgAl-LDHs (1:2) revealed the presence of Mg, Al, O, and Cu elements.

**Figure 4:**
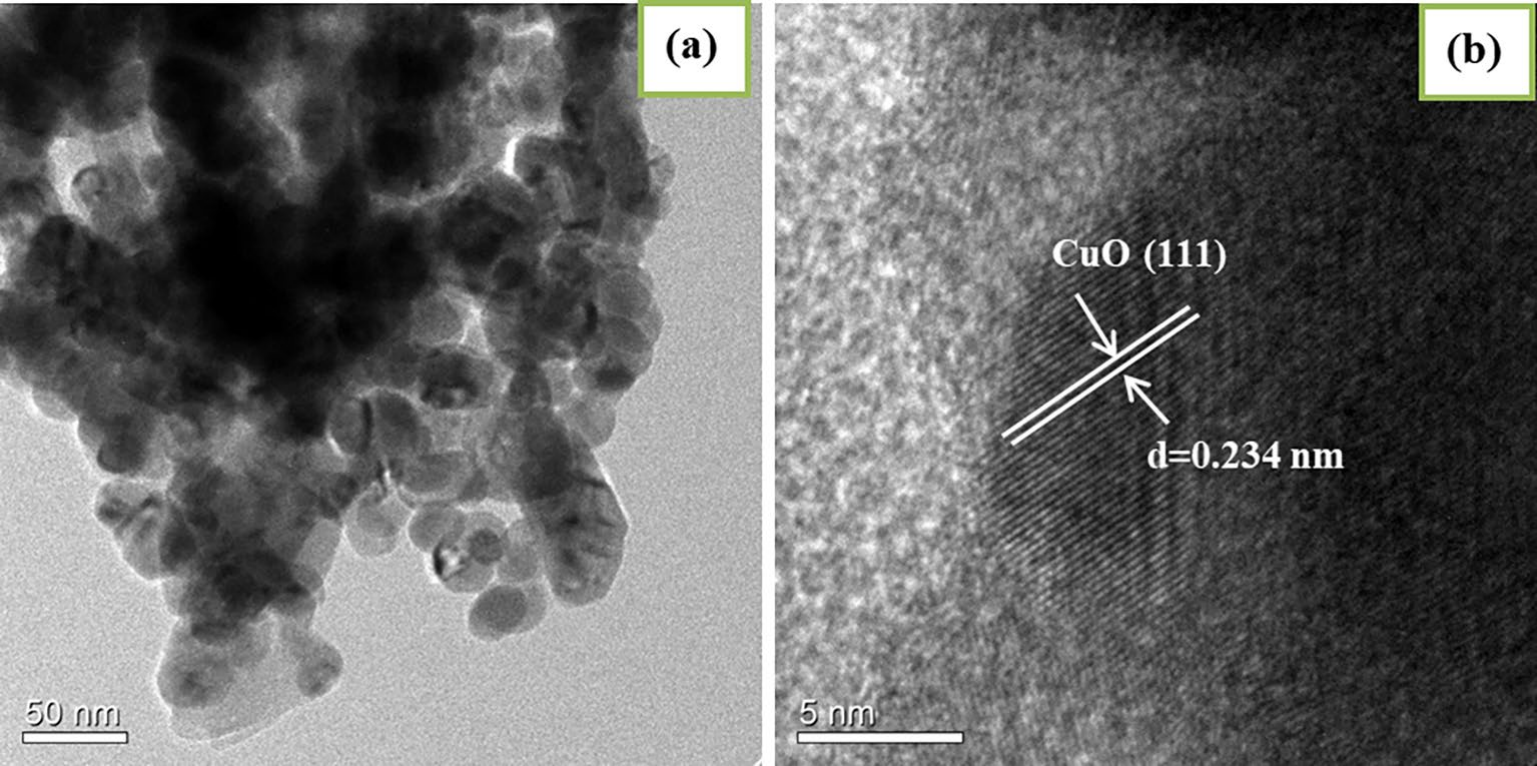
(**a**) TEM, and (**b**) HRTEM images of CuO/MgAl LDHs (1:2) nanocomposites.

To understand the morphology and microstructure of the sample in detail, the TEM analysis was performed. The TEM and HRTEM of the CuO/MgAl-LDHs (1:2) nanocomposite are indicated in Fig. 4. TEM image confirms that dark spots of CuO nanoparticles are distributed in the matrix of LDHs (Fig. 4a). Moreover, the d-spacing of the particles adhered to the surface of the LDH support is 0.234 nm, as shown from the HRTEM image (Fig. 4b), which belongs to the CuO (111) plane. The results revealed that the composites of the nanosized CuO-LDHs matrix were formed. Zeta potential (ZP) measurements were also performed to check the stability of the particles suspensions and their surface charge properties^59^. The average zeta-potential values for CuO, MgAl-LDHs, and CuO/MgAl-LDHs (1:2) catalysts were + 15.45 mV, + 32.7 mV, and + 19.75 mV, respectively, and shown in Fig. 1S (Supplementary). The lower dispersions ZP values will a tendency of coagulation, aggregation, or flocculation due to van der Waals interparticle attraction^60^. After CuO was incorporated into LDHs, the dispersion of ZP f was changed to + 19.75 mV, indicating that the CuO particles were well dispersed into MgAl-LDHs matrix which is also confirmed TEM analysis (Fig. 4).

**Figure 5:**
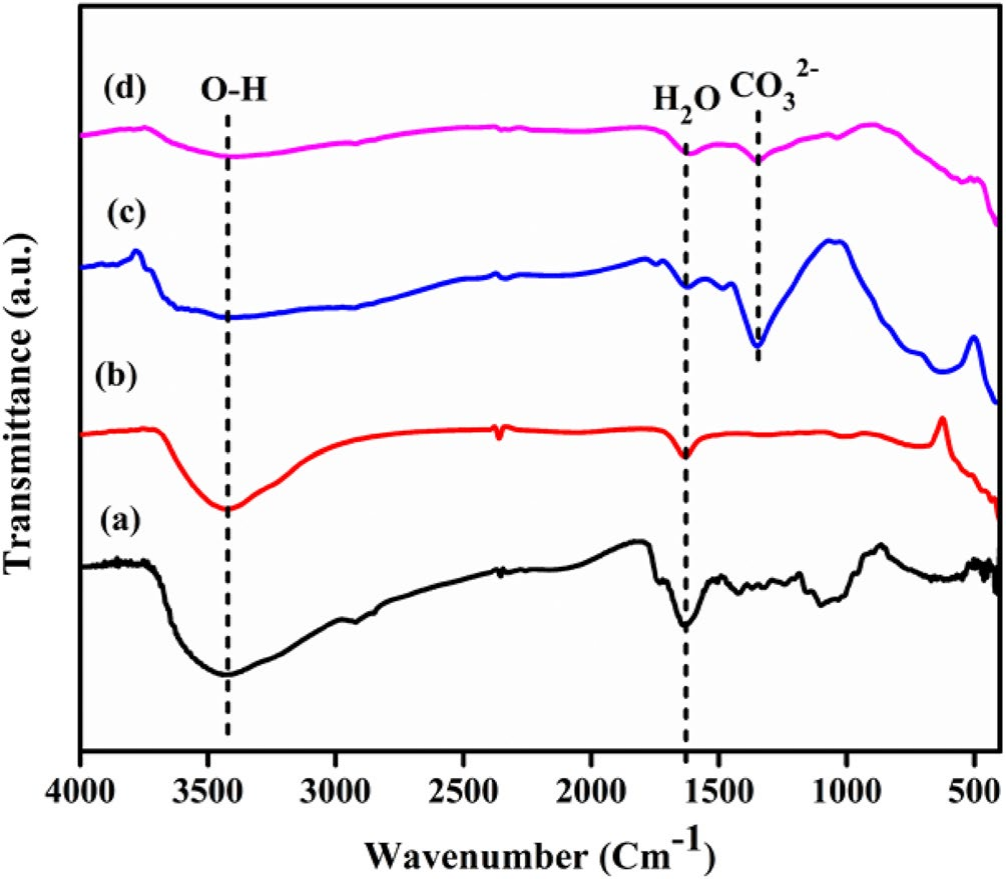
FTIR spectra of: (**a**) *Allium cepa* L. peels extract, (**b**) CuO NPs, (**c**) MgAl-LDHs, and (**d**) CuO/MgAl-LDHs (1:2).

The functional groups of the as-prepared CuO NPs, MgAl-LDHs, and CuO/MgAl LDHs (1:2) samples were investigated by FTIR spectroscopy. As shown in Fig. 5a,b, the O–H stretching vibrations were represented by an absorption band at 3427 cm^−1^. An asymmetric stretching of C–O occurs at 1244 cm^−1^ resulting in cyclic polyphenol compounds^61^. In Fig. 5c, the stretching modes of the O–H groups associated with metal cation bonds in the hydroxide layer and the stretching vibrations of interlayer water molecules, attributed to the strong and broad absorption peak at 3440 cm^−1^ for pure MgAl-LDHs^42^. The asymmetric vibration of CO_3_^2−^ anions and the bending vibration of water molecules between layers in the interlayer region affected the peaks at 1345 cm^−1^ and 1622 cm^−1^, respectively, while the presence of C–O and C=O bonds might cause the CO_3_^2−^ peak to break^35,62^. The vibrations of M–O/M–O–M (M = Mg, Al) characteristic of the double-lamellar structure are responsible for the remaining bands below^63^ 1000 cm^−1^. The CuO/MgAl-LDHs (1:2) spectrums comprised all feature bands of CuO NPs and MgAl-LDHs, as shown in Fig. 5d.

**Figure 6:**
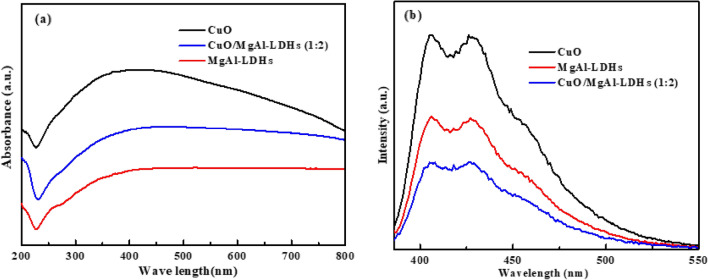
(**a**) UV–vis absorption and (**b**) PL spectra of CuO NPs, MgAl-LDHs, and CuO/MgAl-LDHs (1:2).

The optical properties of the CuO NPs, MgAl-LDHs, and CuO/MgAl-LDHs (1:2) samples were examined and shown in Fig. 6a. The absorbance of the MgAl-LDHs was lower than that of CuO and CuO/MgAl-LDHs (1:2). However, the absorbances of the CuO NPs and CuO/MgAl-LDHs samples were higher as compared to bare MgAl-LDHs. The results indicated that the enhanced absorption of the CuO/MgAl-LDHs composite sample could be due to the visible light- responsive property of CuO. Although CuO had higher absorbance in the visible light region, the photocatalytic performance could be lower due to the higher electron and hole recombination rates. However, significant adsorption intensity enhancement of the MgAl-LDHs may be attributed to the quantum effect of CuO which will inhibit the electron–hole pair’s recombination rate that facilitates the degradation efficiency.

The PL emission spectra of CuO NPs, MgAl-LDHs, and CuO/MgAl-LDHs (1:2) composite were performed at 300 nm exited energy and are shown in Fig. 6b. The PL emission peak for the CuO/MgAl-LDHs (1:2) is substantially reduced, indicating a higher separation efficiency of photoinduced charge carriers than bare samples^54^. The rate of the recombination of excited electron–hole pairs determines the intensity of PL emission. The higher intensity indicates a faster recombination rate, while the lower intensity indicates a large amount of transferred or trapped electrons in which the recombination rate is suppressed. The CuO/MgAl-LDHs (1:2) composite had much lower emission intensity than CuO NPs and MgAl-LDHs bare samples indicating that the recombination of the photogenerated electron–hole pairs was effectively inhibited. The inhibition of the electron–hole pairs facilitates the degradation of organic pollutants^64^.

### Photocatalytic activities

**Figure 7:**
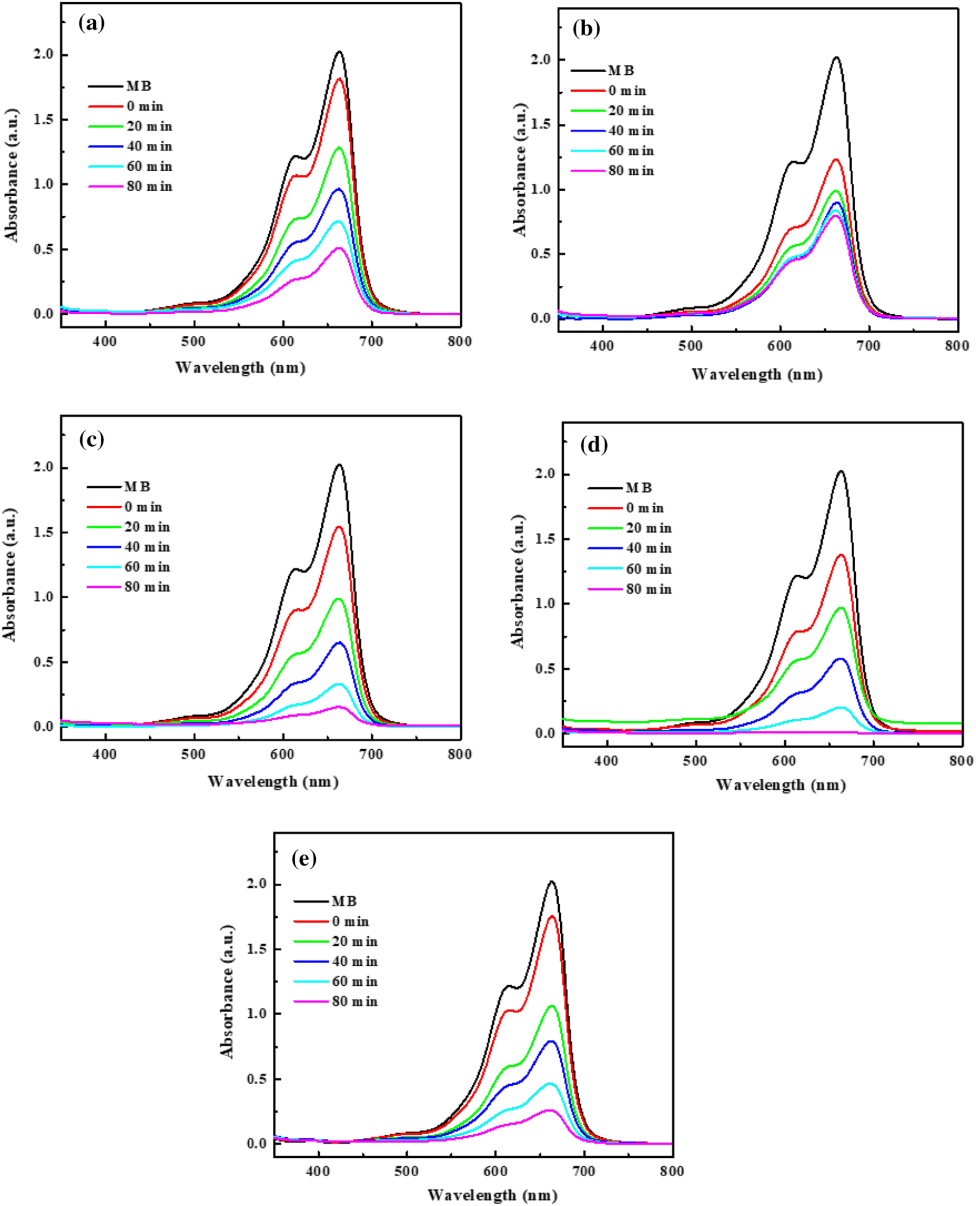
UV–vis absorption spectra for MB degradation with (**a**) CuO NPs, (**b**) MgAl-LDHs, (**c**) CuO/MgAl-LDHs (1:1), (**d**) CuO/MgAl-LDHs (1:2), and (**e**) CuO/MgAl-LDHs (2:1).

The photocatalytic performances of the CuO NPs, MgAl-LDHs, CuO/MgAl-LDHs (1:1), CuO/MgAl-LDHs (1:2), and CuO/MgAl-LDHs (2:1) composite samples were tested in the degradation of MB dye. The UV–vis absorption spectra for the MB degradations are shown in Fig. 7. As demonstrated in Fig. 7a–e, the time-dependent dye absorption intensity was decreased after the photocatalytic reaction was performed. The higher photocatalytic performance was observed in the presence of CuO/MgAl-LDHs (1:2) sample than other samples within 80 min irradiation time. The results also indicated that the addition of the optimum amounts of CuO in the composite preparation system can affect the photocatalytic properties. It can be also demonstrated that the composite catalyst showed better performances than bare CuO NPs and MgAl-LDHs samples. It could be due to interfacial charge transfers which facilitate the electron migration and suppression of electron and hole recombination^41^.

**Figure 8:**
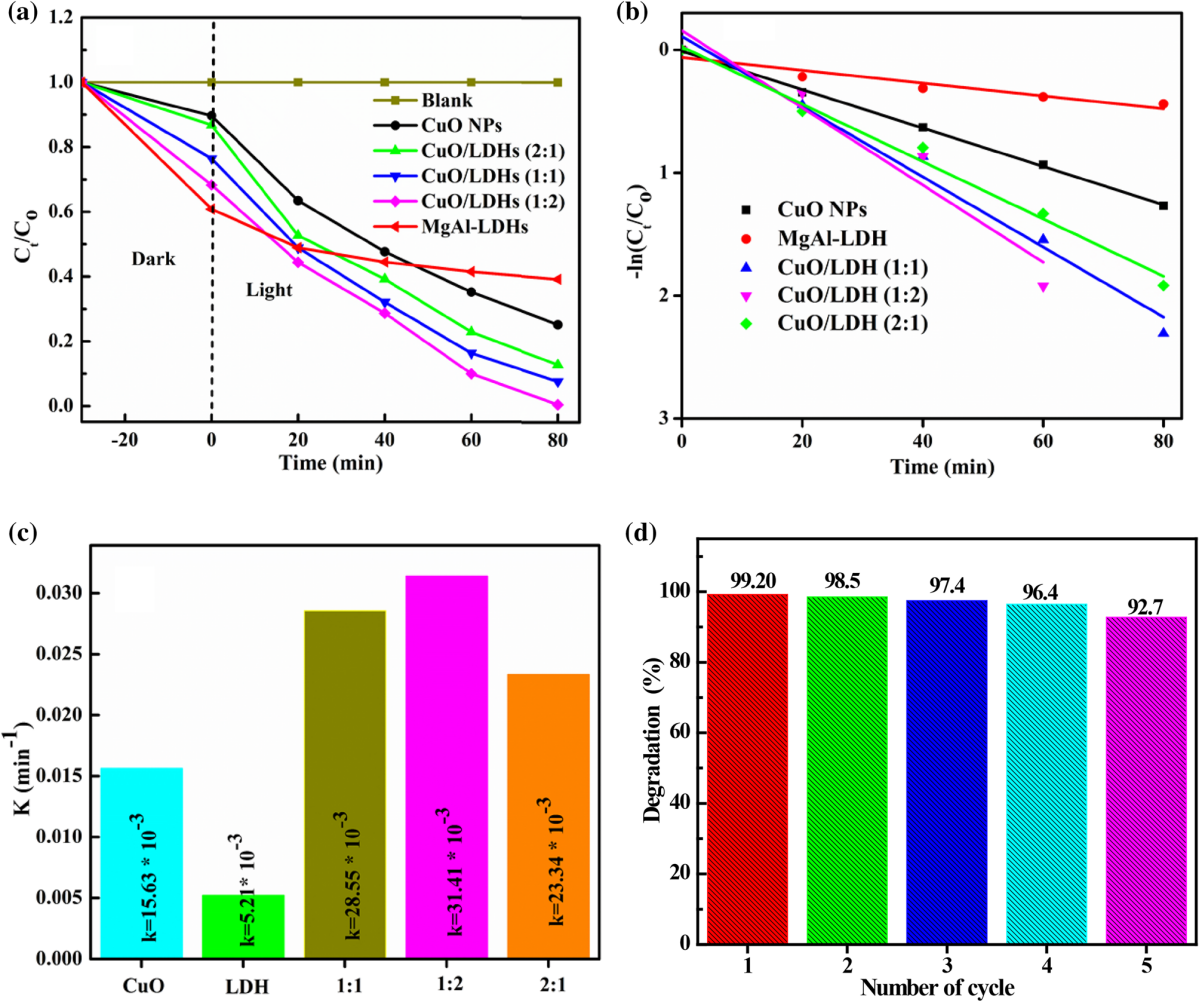
(**a**) The C/C_0_ versus irradiation time plot of the MB degradation with different catalysts, (**b**,**c**) the kinetics study of different catalysts, and (**d**) the stability of CuO/MgAl-LDHs (1:2) sample.

Moreover, the degradation performance was also demonstrated as shown in Fig. 8a. The degradation of MB was almost negligible throughout the catalyst (blank). The photodegradation efficiency of MB with CuO/MgAl-LDHs (1:2) was 99.20%. However, the MgAl-LDHs, CuO NPs, CuO/MgAl-LDHs (2:1), and CuO/MgAl-LDHs (1:1) catalyst degradation efficiencies were 35.53, 71.87, 85.30, and 90.03%, respectively. Furthermore, the MgAl-LDHs demonstrated high adsorption of MB molecules in the dark. It can be attributed to MgAl LDHs having a large surface area and low visible light activity. Zhou et al. discovered similar results of MgAl-LDHs^65^. The findings suggest that CuO are critical for photocatalytic activity, and adding CuO NPs into MgAl-LDHs could boost photocatalytic activity.

Figure 8b,c provides detailed information on the kinetic parameters of pseudo-first-order fitting for MB degradation^66^. The degradation rate constant (k) for CuO/MgAl-LDHs (1:2) was estimated to be 0.0314 min^−1^, while the kinetic rates for CuO, LDHs, CuO/MgAl-LDHs (2:1), and CuO/MgAl-LDHs (1:1) samples were 0.0156, 0.0052, 0.0234, and 0.02855 min^−1^, respectively. The CuO/MgAl-LDHs (1:2) kinetic value was higher than that of all other samples. The findings showed that the CuO/MgAl-LDHs (1:2) composite had the best photocatalytic activity in visible light irradiation. Moreover, the catalytic performance of the composite catalyst was also compared with other literature reports. As it is demonstrated in Table S1 (Supplementary), the CuO/MgAl-LDHs (1:2) composite catalyst was comparable with other catalysts in the degradation of organic pollutants. Figure 8d also demonstrates the stability of the CuO/MgAl-LDHs (1:2) composite catalyst. The CuO/MgAl-LDHs (1:2) composite catalyst showed higher photocatalytic stability after five cycles. The slightly reduced activity was primarily due to the mass loss of the photocatalyst during centrifugation to collect the reused catalyst. After five cycles, 92.7% of the MB dye degradation was maintained.

**Figure 9:**
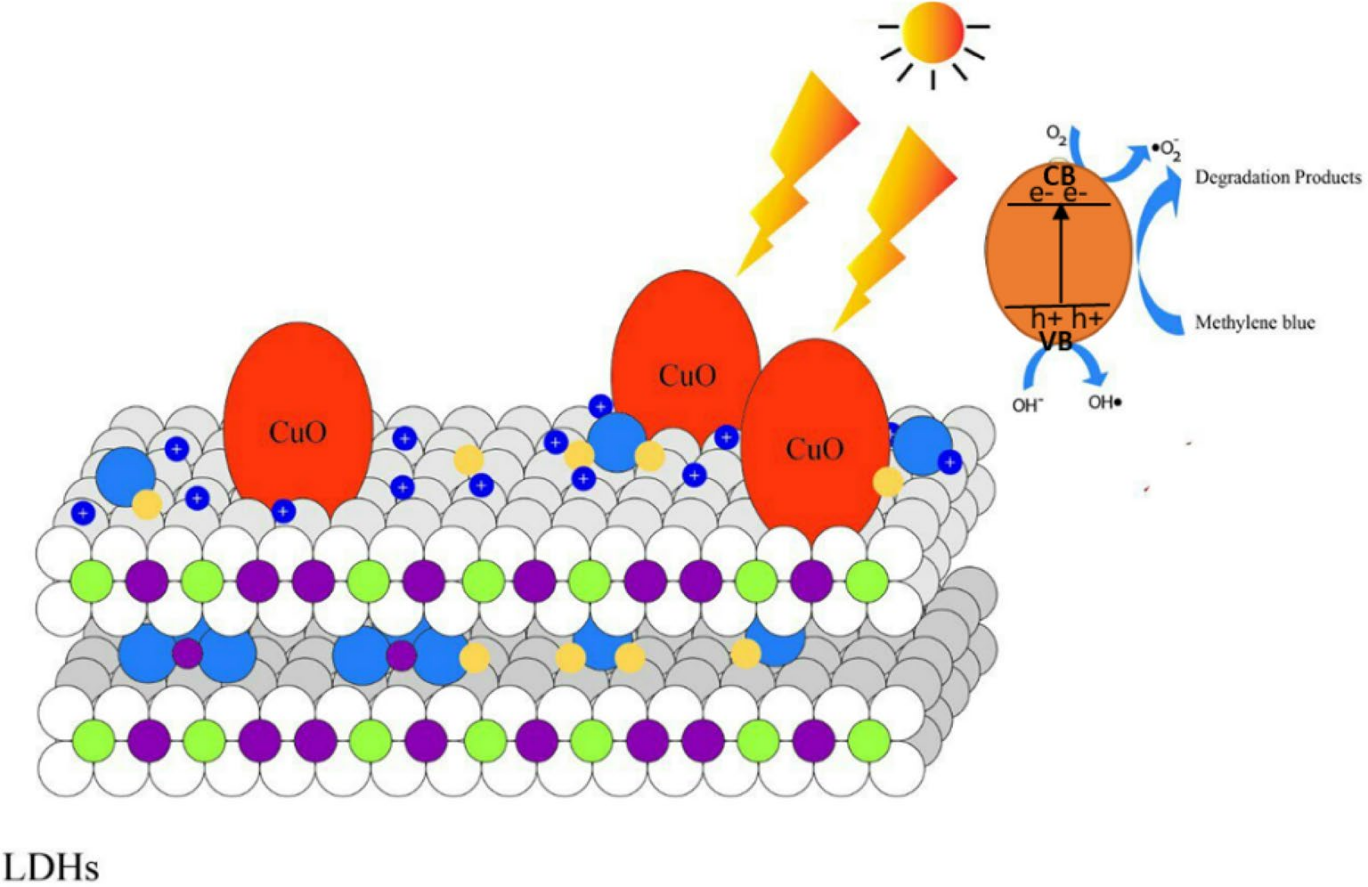
The proposed photocatalytic degradation mechanism of MB with CuO/MgAl-LDHs composites catalyst.

The photocatalytic degradation mechanism was proposed according to the experimental findings and characterizations results. When the photon energy which is equal to or greater than the band gap energy strikes the surface of the CuO/MgAl-LDHs, the electrons will be ejected from the balance band and moved to the conduction band^67^. The CuO creates electron–hole pairs and the positive charges on the surface of LDHs platelets can attract electrons (e^−^) produced by the CuO particles, while the holes (h^+^) move to the opposite direction, leading to the separation of the photogenerated e^−^ and h^+^ pairs^34^. The photogenerated holes (h^+^) reacted with hydroxyl ions to produce hydroxyl radicals (·OH), and the ·OH radicals will oxidize the organic contaminants^68^. Furthermore, the LDH layer's surface ^**−**^OH groups reacted with valence band holes to produce hydroxyl radicals (·OH), which are significant variables in photo-oxidation reactions^39^. Similarly, the holes can directly interact with organic pollutants and degradation will be facilitated. According to the experimental finding of scavenging effects (Fig. S2) (Supplementary), the degradation efficiency in the presence of isopropanol (·OH scavenger) significantly decreased. However, in the presence of EDTA-2Na (h^+^ scavenger) and AgNO_3_ (e^−^ scavenger), the degradation efficiencies were better than in the presence isopropanol. As it was demonstrated from trapping experiments, the catalyst performance without scavengers was much higher than that of the degradation in the presence of scavengers. Simultaneously, superoxide radicals (^·^O_2_^**−**^) were formed when excited electrons were interacted by dissolved oxygen species in an aqueous solution^67^. Organic matter could be decomposed into CO_2_ and H_2_O after interaction with reactive h^+^, ^·^O_2_^−^ and ·OH species^69^. Therefore, the ·OH, ^·^O_2_^**−**^, h^+^, e^−^ reactive species can be responsible for the degradation of MB dye using CuO/MgAl-LDHs nanocomposites. Moreover, the CuO NPs, which are effectively distributed over the LDH and their synergistic impact in quick dye adsorption followed by rapid photodegradation are attributed to the photocatalytic improvement of the CuO/MgAl-LDHs nanocomposites. It should be highlighted that the strong interaction with LDH aided charge transport and improved the photocatalytic function of composites. The possible degradation mechanism is depicted in Fig. 9.

## Conclusions

The CuO semiconductor catalyst synthesized with a green method was combined with MgAl-LDH and applied for the degradation of MB dye. The catalytic performance of the CuO/MgAl-LDHs (1:2) composite exhibited the highest catalytic performance and degraded 99.20% MB dye within 80 min. However, only 90.03, 85.30, 71.87, and 35.53% of MB dye degradation was achieved by CuO/MgAl-LDHs (1:1), CuO/MgAl-LDHs (2:1), CuO NPs, and MgAl-LDHs catalysts, respectively. The enhanced photocatalytic activity was attributed to a synergistic effect, homogeneous dispersion of CuO NPs, adsorption of MB on MgAl-hydroxyl-rich LDH's structure, and lower band gap of CuO NPs. The catalyst stability was also checked and 92.7% of the MB dye removal efficiency was still maintained after five cycles. Therefore, the CuO/MgAl-LDHs-based photocatalyst could be a potential candidate for the environmental remediation.

### Supplementary Information

Supplementary Information.

## Data Availability

All data generated or analyzed during this study are included in this published article.
