# Supplementary material for: Photocatalytic activity of the biogenic mediated green synthesized CuO nanoparticles confined into MgAl LDH matrix
Source: Sci Rep. 2024 Jan 28;14:2314. doi: 10.1038/s41598-024-52547-w (PMC10822861; doi:10.1038/s41598-024-52547-w)
Supplement: Supplementary file 1 — Supplementary Information. [file 41598_2024_52547_MOESM1_ESM.docx]

**Supplementary Information**

# Photocatalytic activity of the biogenic mediated green synthesized CuO nanoparticles confined in to MgAl LDH matrix

Hildana Tesfaye Berede^1^, Dinsefa Mensur Andoshe^1^, Noto Susanto Gultom^2^, Dong-Hau Kuo^2^, Xiaoyun Chen^3^, Hairus Abdullah^2^, Tadele Hunde Wondimu^1^, Yi-nan Wu^4,5^, Osman Ahmed Zelekew^1,*^

^1^Department of Materials Science and Engineering, Adama Science and Technology University, Adama, Ethiopia

^2^Department of Materials Science and Engineering, National Taiwan University of Science and Technology, Taipei 10607, Taiwan

^3^College of Materials Engineering, Fujian Agriculture and Forestry University, Fuzhou 350002, China

^4^College of Environmental Science and Engineering, State Key Laboratory of Pollution Control and Resource Reuse, Tongji University, 1239 Siping Rd., Shanghai 200092, China

^5^Shanghai Institute of Pollution Control and Ecological Security, 1239 Siping Rd., Shanghai 200092, China

*Corresponding author: osman.ahmed@astu.edu.et; osmax2007@gmail.com

**Supplementary Figures**

Figure 1S. Zeta potential analysis of synthesized (a) CuO NPs, (b) MgAl-LDHs and (c) CuO NPs/ MgAl-LDHs (1:2)

Figure S1. Trapping experiments of the CuO NPs/MgAl-LDHs (1:2) composite catalyst.

**Supplementary Table**

| Table S1. Comparison of the photocatalytic degradation performance of the CuO/MgAl-LDHs (1:2) composite with other reported catalysts. | | | | | |
| --- | --- | --- | --- | --- | --- |
| Catalysts | Catalyst (mg) | [MB] (mg L^-1^) | Degradation (%) | Time (min) | Ref. |
| CuO NPs | 20 | 30 | 89 | 150 | [1] |
| g-C_3_N_4_/ZnO/Mg-Al LDH | - | - | 96.5 | 115 | [2] |
| Ag-Si_NWs_-Cu_NPs_ | - | 20 | 92 | 150 | [3] |
| TiO_2_/MgZnAl-5 | 300 | 50 | ~100 | 360 | [4] |
| CuO-SWCNT-5 | 150 | 100 | 97.33 | 120 | [5] |
| Cu_2_O/CLDH-773 | 400 | 10 | 86.2 | 360 | [6] |
| CuO/MgAl-LDH (1:2) | 25 | 10 | 99.20 | 80 | **This work** |

**Reference**

[1] S. Sathiyavimal, S. Vasantharaj, V. Veeramani, M. Saravanan, G. Rajalakshmi, T. Kaliannan, F.A. Al-Misned, A. Pugazhendhi, Green chemistry route of biosynthesized copper oxide nanoparticles using Psidium guajava leaf extract and their antibacterial activity and effective removal of industrial dyes, Journal of Environmental Chemical Engineering 9(2) (2021) 105033.

[2] K. Bhuvaneswari, G. Palanisamy, T. Pazhanivel, T. Maiyalagan, G. Bharathi, Photodegradation Activity of Nitrogen‐rich Graphitic Carbon Nitride Intercalated ZnO\Mg‐Al Layered Double Hydroxide Ternary Nanocomposites on Methylene Blue Dye, ChemistrySelect 4(11) (2019) 2982-2990.

[3] M.R.G. Robles, J.d.J.P. Bueno, C.S.A. Syllas, M.L.M. López, F.M. Guerrero, Silver/Silicon nanowires/copper nanoparticles heterojunction for methyl orange degradation by heterogeneous photocatalysis under visible irradiation, MRS Advances 3(64) (2018) 3933-3938.

[4] M.F. de Almeida, C.R. Bellato, A.H. Mounteer, S.O. Ferreira, J.L. Milagres, L.D.L. Miranda, Enhanced photocatalytic activity of TiO2-impregnated with MgZnAl mixed oxides obtained from layered double hydroxides for phenol degradation, Applied Surface Science 357 (2015) 1765-1775.

[5] K.P. Sapkota, I. Lee, M.A. Hanif, M.A. Islam, J. Akter, J.R. Hahn, Enhanced visible-light photocatalysis of nanocomposites of copper oxide and single-walled carbon nanotubes for the degradation of methylene blue, Catalysts 10(3) (2020) 297.

[6] Y. Zhou, W. Hu, J. Yu, F. Jiao, Effective photocatalytic degradation of methylene blue by Cu 2 O/MgAl layered double hydroxides, Reaction Kinetics, Mechanisms and Catalysis 115 (2015) 581-596.
